# Phase 2 Study of Zilovertamab Vedotin in Participants with Metastatic Solid Tumors

**DOI:** 10.1158/2767-9764.CRC-25-0019

**Published:** 2025-09-17

**Authors:** Funda Meric-Bernstam, Martin Gutierrez, Enrique Sanz-Garcia, Diego Villa, Jun Zhang, Jennifer Friedmann, Fengting Yan, Mark A. Socinski, John Sarantopoulos, Luis E. Raez, Quincy S. Chu, Maxime Chénard-Poirier, Manash S. Chatterjee, Hong Ren, Qi Liu, Douglas A. Levine, Komal L. Jhaveri

**Affiliations:** 1 Department of Investigational Cancer Therapeutics, Division of Cancer Medicine, The University of Texas MD Anderson Cancer Center, Houston, Texas.; 2 Hackensack University Medical Center, Hackensack, New Jersey.; 3 Division of Medical Oncology and Hematology, Princess Margaret Cancer Centre, Toronto, Canada.; 4 University of British Columbia, BC Cancer Centre for Lymphoid Cancer, Vancouver, Canada.; 5 Division of Medical Oncology, Department of Internal Medicine, University of Kansas Medical Center, Kansas City, Kansas.; 6 Division of Medical Oncology, Department of Cancer Biology, University of Kansas Medical Center, Kansas City, Kansas.; 7 Gerald Bronfman Department of Oncology, Jewish General Hospital, Montreal, Canada.; 8 Swedish Cancer Institute, Seattle, Washington.; 9 AdventHealth Cancer Institute, Orlando, Florida.; 10 Institute for Drug Development, Mays Cancer Center at University of Texas Health San Antonio MD Anderson Cancer Center, San Antonio, Texas.; 11 Memorial Cancer Institute/Florida Atlantic University (FAU), Miami, Florida.; 12 Division of Medical Oncology, Department of Oncology, Cross Cancer Institute, Edmonton, Canada.; 13 CHU de Québec-Université Laval, Québec, Canada.; 14 Merck & Co., Inc., Rahway, New Jersey.; 15 Memorial Sloan Kettering Cancer Center, New York, New York.; 16 Weill Cornell Medical College, New York, New York.

## Abstract

**Purpose:**

Zilovertamab vedotin, an antibody–drug conjugate targeting receptor tyrosine kinase–like orphan receptor 1 (ROR1), had manageable safety and promising antitumor activity in participants with relapsed or refractory non–Hodgkin lymphomas. We evaluated zilovertamab vedotin in participants with previously treated metastatic solid tumors.

**Patients and Methods:**

This phase 2, open-label, nonrandomized study (NCT04504916) enrolled participants with metastatic triple-negative breast cancer, hormone receptor–positive breast cancer, nonsquamous non–small-cell lung cancer, platinum-resistant ovarian cancer, or pancreatic cancer. Participants received zilovertamab vedotin ≤2.5 mg/kg once every 3 weeks (Q1/3W) or <1.75 mg/kg twice every 3 weeks (Q2/3W). The primary endpoint was objective response rate per RECIST version 1.1 by blinded independent central review. ROR1 protein expression was correlated with clinical outcomes.

**Results:**

A total of 102 participants were enrolled (Q1/3W, *n* = 70; Q2/3W, *n* = 32). The objective response rate was 1% [95% confidence interval (CI), 0%–8%] with Q1/3W dosing (one partial response, hormone receptor–positive/HER2-negative breast cancer cohort) and 0% with Q2/3W dosing. The median progression-free survival (95% CI) was 2.3 (2.0–4.1) and 1.9 (1.7–2.1) months, respectively; the median overall survival (95% CI) was 8.3 (5.2–10.3) and 5.5 (4.4–11.0) months, respectively. Across dosing regimens, treatment-related adverse events were reported in 85 participants (83%), most commonly fatigue (29%) and nausea (28%). Treatment-related peripheral neuropathy occurred in 8%. Treatment-related adverse events led to dose interruption/reduction in 32 participants (31%) and permanent treatment discontinuation in 7 (7%). Tissue for ROR1 IHC was available on 17 participants, with only 3 (all nonresponders) showing ROR1 expression.

**Conclusions:**

Zilovertamab vedotin had minimal antitumor activity, with only a single responder, and manageable safety in participants with previously treated metastatic solid tumors.

**Significance:**

Zilovertamab vedotin had minimal antitumor activity and manageable safety in participants with previously treated metastatic solid tumors of various histologic subtypes. The results suggest that further development of zilovertamab vedotin in these solid tumors is not warranted.

## Introduction

Receptor tyrosine kinase–like orphan receptor 1 (ROR1) is a type 1 transmembrane protein that shares homology with other receptor tyrosine kinases and shows high evolutionary conservation (1, 2). High levels of *ROR1 *gene and protein expression have been reported in multiple solid tumors, including breast, lung, ovarian, and pancreatic carcinomas, as well as in lymphoid and myeloid malignancies (3–5). The activation of ROR1 has been associated with signaling pathways that are conducive to tumor growth, which has attracted interest as a potential anticancer target (6).

Zilovertamab vedotin (MK-2140) is an ROR1-targeting antibody–drug conjugate (ADC) composed of zilovertamab conjugated to vedotin, which is a combination of the linker maleimidocaproyl–valine–citrulline–para–aminobenzoate and the antimicrotubule cytotoxin monomethyl auristatin E (MMAE; ref. 7). The anticancer activity of zilovertamab vedotin is primarily due to binding of the ADC to ROR1-expressing tumor cells, internalization of the ADC–ROR1 complex with trafficking to tumor cell lysosomes, and release of MMAE via lysosomal proteolytic cleavage (8, 9). The binding of free MMAE to tubulin disrupts the microtubule network within the tumor cell, inducing cell-cycle arrest and apoptotic tumor cell death (8).

In preclinical studies, the humanized IgG1 mAb zilovertamab (previously known as UC-961 or cirmtuzumab) inhibited proliferation of ovarian and endometrial cancer cells (10, 11). Preclinical findings also showed that zilovertamab vedotin induced tumor shrinkage in mouse models of human lymphoid cancers (8, 9, 12). In a phase 1 study, zilovertamab (doses ranging from 15 µg/kg to 20 mg/kg) inhibited ROR1 signaling in participants with chronic lymphocytic leukemia; none of the 22 evaluable participants achieved a complete or partial response, although participants experienced a greater trend for reduction in disease burden with higher doses of zilovertamab (13). Preliminary findings in participants with relapsed or refractory non–Hodgkin lymphoma demonstrated that zilovertamab vedotin 2.5 mg/kg had a tolerable safety profile and promising antitumor activity [objective response rate (ORR), 30%–42%; refs. 7,14,15], with exposure-dependent targeting of ROR1 (16). There are no reports on the use of zilovertamab vedotin monotherapy in participants with solid tumors. This phase 2 study was designed to evaluate the efficacy, safety, tolerability, and pharmacokinetics of zilovertamab vedotin in participants with previously treated metastatic solid tumors.

## Materials and Methods

The study (ClinicalTrials.gov, NCT04504916) was conducted according to International Council for Harmonization Good Clinical Practice guidelines and followed all local laws and regulations. The protocol (MK-2140-002) and associated amendments were reviewed and approved by the local Institutional Review Board or independent Ethics Committee. All participants provided written informed consent before any study procedures were performed.

### Participants

Eligible participants were ages ≥18 years, with a histologically or cytologically confirmed diagnosis of triple-negative breast cancer (TNBC), hormone receptor–positive/HER2-negative (HR+/HER2−) breast cancer, nonsquamous non–small-cell lung cancer (NSCLC), gastric cancer, pancreatic cancer, or platinum-resistant ovarian cancer. Other key inclusion criteria were metastatic disease that progressed during or following previous treatment, radiographically measurable disease as determined by the investigator, an Eastern Cooperative Oncology Group (ECOG) performance status of 0 or 1, an available pretreatment tumor tissue biopsy (fresh or archival), completion of all previous anticancer therapy ≥2 weeks before the start of study treatment, life expectancy of ≥3 months from enrollment, and adequate organ function. Major exclusion criteria included grade >1 peripheral neuropathy, central nervous system malignancy, another major cancer with disease manifestations or therapy that could adversely affect participant safety or compromise interpretation of study results, uncontrolled ongoing infection, significant cardiovascular disease, and liver cirrhosis. Central ROR1 expression was not a prerequisite to participating in the study.

### Design and treatment

In this phase 2, open-label, nonrandomized, single-arm, multisite study, the initial starting dose of zilovertamab vedotin was 2.5 mg/kg administered intravenously on day 1 of repeated 21-day cycles [once every 3 weeks (Q1/3W)]. However, emerging serum pharmacokinetic data in participants with hematologic malignancies indicated that the ADC was eliminated quickly [elimination half-life (t_1/2_), ∼4 days] and high levels of receptor occupancy could not be maintained for the duration of the 3-week cycle. Similar findings were observed based on preliminary serum pharmacokinetic data in participants with solid tumors. The initial starting dose of zilovertamab vedotin was therefore altered via a protocol amendment to 1.75 mg/kg intravenous dosing (1.5 mg/kg intravenously for participants concurrently receiving a strong cytochrome P450 3A4 inhibitor) on days 1 and 8 of repeated 21-day cycles (Q2/3W; Supplementary Table S1), aiming for greater tumor exposure. There were plans to increase the initial starting dose via another protocol amendment to 2.0 mg/kg for the Q2/3W regimen, but the amendment was not activated, and participants never enrolled at this dose because of study termination. Dosing modifications were permitted based on toxicities. Study treatment continued until progressive disease, unacceptable toxicity, intercurrent illness, investigator decision, noncompliance, or participant withdrawal of consent. The study design is depicted in Supplementary Fig. S1.

### Endpoints and assessments

The primary endpoint of this study was the confirmed ORR per RECIST (RRID: SCR_026435) version 1.1 as assessed by blinded independent central review (BICR). For this endpoint, participants had to achieve a complete or partial response that was confirmed ≥4 weeks after the first indication of a response was observed. Tumor burden was assessed by medical imaging, preferably using CT or MRI, at screening, every 9 weeks up to 36 weeks, and every 12 weeks thereafter.

Secondary endpoints included confirmed ORR per RECIST version 1.1 as assessed by the investigator, time to response (TTR), duration of response (DOR), progression-free survival (PFS), time to treatment failure (TTF), overall survival (OS), safety, and pharmacokinetics. TTR was defined as the interval from the initiation of zilovertamab vedotin to the first documentation of objective tumor response. DOR was defined as the interval between the first documentation of objective tumor response and progressive disease or death from any cause. PFS was the interval from the initiation of zilovertamab vedotin to the first documentation of progressive disease or death from any cause. TTF was the interval from the initiation of zilovertamab vedotin to the first documentation of progressive disease, permanent discontinuation of zilovertamab vedotin because of an adverse event (AE), or death from any cause. OS was defined as the interval from the initiation of zilovertamab vedotin to death of any cause. AEs were monitored throughout the study and for 30 days after treatment and were graded by the investigator according to the NCI Common Terminology Criteria for Adverse Events (RRID: SCR_010296) version 5.0. Pharmacokinetic endpoints included maximum plasma concentration (C_max_), time to C_max_ (T_max_), t_1/2_, and area under the plasma concentration–time curve (AUC) as calculated using noncompartmental methods for total zilovertamab vedotin, total antibody, and MMAE. Samples for pharmacokinetic analyses were obtained during cycle 1. In participants receiving Q1/3W dosing, samples were obtained before dosing, at the end of infusion, and at 2, 4, 168, 336, and 504 (predose in cycle 2) hours after the day 1 infusion. In participants receiving Q2/3W dosing, samples were obtained before dosing and at 0.5 and 168 hours after the day 1 infusion and before dosing and at 0.5, 168, and 336 (predose in cycle 2) hours after the day 8 infusion.

### Sample collection and IHC analysis

Formalin-fixed and paraffin-embedded pretreatment tumor tissue was collected from either a fresh tumor biopsy or an archival sample obtained since the last prior treatment and within 24 weeks before the start of study treatment. No posttreatment biopsies were requested or obtained. Due to the limited ORR and the availability of biospecimens, two cohorts were empirically created to represent participants with shorter and longer durations of treatment. The short treatment cohort was defined as participants who progressed at the first interval tumor assessment (9-week on-study radiology assessment after three cycles of treatment), and the longer treatment cohort was defined as participants who remained on study treatment for at least six cycles of therapy.

Formalin-fixed and paraffin-embedded samples sectioned at 5 µm were baked at 60°C for 45 minutes and deparaffinized in a xylene to alcohol to water gradient using the Leica Autostainer XL (Leica Biosystems). Sections were subjected to heat-induced antigen retrieval in Target Retrieval Solution (ethylenediaminetetraacetic acid, pH 8.0, for ROR1 and Target Retrieval Solution, pH 6.1, for marker of proliferation Ki-67) at 120°C for 4 minutes at 10 to 15 psi. Endogenous peroxidase was quenched by incubating the slides in 3% hydrogen peroxide for 10 minutes. The following staining procedure was performed in the Autostainer 480S (Thermo Fisher Scientific). Slides were incubated with the primary antibody, rabbit anti-human ROR1 clone (S67H1L31, Roche Tissue Diagnostics, 7 µg/mL), or mouse anti-human Ki-67 clone (M7240, Dako, 0.7 µg/mL) for 60 minutes. The ROR1 clone chosen was the best performing from several rounds of custom antibody generation and assessment of numerous commercially available clones. Slides were then incubated with a secondary antibody (Envision rabbit horseradish peroxidase for ROR1 and Envision mouse horseradish peroxidase for Ki-67) for 30 minutes. Slides were rinsed in TBS with Tween 20 between each staining step. 3,3′-diaminobenzidine chromogen was used to visualize antibody binding, and an enhancer was used for ROR1 to increase the 3,3′-diaminobenzidine signal. Stained slides were counterstained with Mayer’s hematoxylin solution and bluing reagent and then cover-slipped for review by a pathologist.

ROR1 and Ki-67 protein expression in tumor tissues detected by IHC staining were analyzed and scored by a pathologist using the *H*-score system (score, 0–300) that was calculated using the following formula: *H*-score = (percentage of tumor cells stained at weak staining intensity + percentage of tumor cells stained at moderate intensity × 2 + percentage of tumor cells stained at strong intensity × 3) × 100. Both cytoplasmic and membranous staining patterns were considered.

### Statistical analysis

It was estimated that approximately 30 participants per disease cohort would need to be accrued for enrollment to proceed through both stages of the Simon 2-stage enrollment paradigm for that cohort, with allowance for five additional participants (approximately 35 total per cohort), as some participants may not be fully evaluable for efficacy. Based on this design, the study had >0.80 power with a significance level of <0.05 when the ORR of a null hypothesis was 5% and the true ORR was 20% for each disease cohort. Efficacy and safety analyses included all participants who received ≥1 dose of zilovertamab vedotin. Pharmacokinetic analyses included all participants who received ≥1 dose of zilovertamab vedotin. ORR point estimates were calculated for each tumor type and dosing schedule together with 95% confidence intervals (CI) using the exact binomial method. Time-to-event endpoints were estimated using the method of Kaplan and Meier with appropriate censoring. The opportunity to conduct an interim analysis was allowed at the sponsor’s discretion, and data were examined on a continuous basis to allow for dose-finding decisions. For each disease cohort, a futility interim analysis was conducted after the first 13 participants had their end-of-cycle 6 radiographic evaluation, without a pause in enrollment.

### Data availability

Merck Sharp & Dohme LLC, a subsidiary of Merck & Co., Inc. (MSD), is committed to providing qualified scientific researchers access to anonymized data and clinical study reports from the company’s clinical trials for the purpose of conducting legitimate scientific research. MSD is also obligated to protect the rights and privacy of trial participants and, as such, has a procedure in place for evaluating and fulfilling requests for sharing company clinical trial data with qualified external scientific researchers. The MSD data-sharing website (available at https://externaldatasharing-msd.com/) outlines the process and requirements for submitting a data request. Applications will be promptly assessed for completeness and policy compliance. Feasible requests will be reviewed by a committee of MSD subject matter experts to assess the scientific validity of the request and the qualifications of the requestors. In line with data privacy legislation, submitters of approved requests must enter into a standard data-sharing agreement with MSD before data access is granted. Data will be made available for request after product approval in the United States and European Union or after product development is discontinued. There are circumstances that may prevent MSD from sharing requested data, including country- or region-specific regulations. If the request is declined, it will be communicated to the investigator. Access to genetic or exploratory biomarker data requires a detailed, hypothesis-driven statistical analysis plan that is collaboratively developed by the requestor and MSD subject matter experts; after approval of the statistical analysis plan and execution of a data-sharing agreement, MSD will either perform the proposed analyses and share the results with the requestor or construct biomarker covariates and add them to a file with clinical data that is uploaded to an analysis portal so that the requestor can perform the proposed analyses.

## Results

### Participants

A total of 102 participants with previously treated metastatic solid tumors were enrolled in the study (Supplementary Fig. S2). Of these participants, 70 (69%) received Q1/3W dosing and 32 (31%) received Q2/3W dosing. At data cutoff (November 15, 2023), all participants had discontinued treatment and long-term follow-up. Participant demographics and baseline characteristics are shown by dosing schedule and cancer type in Table 1. Overall, participants had received a median of 4 (range, 1–21) prior systemic treatment regimens in both the primary and metastatic setting. The median age was 60 (range, 32–88) years, 38% of participants were ages ≥65 years, and the median body mass index was 26.1 (range, 15.6–36.3) kg/m^2^. Most participants were female (86%); 74% were White, 12% were Asian, 8% were Black or African American, and 6% were of other or unknown races. Sixty-six percent of participants had an ECOG performance status of 1. The study population was generally representative of the real-world setting in locations where participants were enrolled (Supplementary Table S2). During the study, participants received a median of 3 (range, 1–18) doses of zilovertamab vedotin over a median of 3 (range, 1–16) cycles. The median follow-up duration was 5.2 (range, 0.4–29.2) months. Study drug exposure and follow-up duration by dosing schedule and cancer type are shown in Supplementary Table S3.

**Table 1.** Participant demographics and baseline characteristics.

​	Q1/3W			Q2/3W			
	TNBC (*n* = 15)	HR+/HER2− (*n* = 35)	NSCLC (*n* = 20)	TNBC (*n* = 11)	NSCLC (*n* = 9)	Ovarian (*n* = 3)	Pancreatic (*n* = 9)
Age, years	​	​	​	​	​	​	​
Median (range)	49 (32–65)	57 (33–75)	71.5 (45–82)	58 (35–70)	68 (49–88)	61 (43–74)	64 (55–78)
≥65, *n* (%)	1 (7)	11 (31)	13 (65)	2 (18)	7 (78)	1 (33)	4 (44)
Sex, *n* (%)	​	​	​	​	​	​	​
Female	15 (100)	34 (97)	15 (75)	11 (100)	7 (78)	3 (100)	3 (33)
Male	0	1 (3)	5 (25)	0	2 (22)	0	6 (67)
Race, *n* (%)	​	​	​	​	​	​	​
White	9 (60)	21 (60)	16 (80)	9 (82)	9 (100)	3 (100)	8 (89)
Asian	3 (20)	8 (23)	0	1 (9)	0	0	0
Black or African American	1 (7)	4 (11)	2 (10)	1 (9)	0	0	0
American Indian or Alaskan Native	1 (7)	0	0	0	0	0	0
Other	0	1 (3)	0	0	0	0	1 (11)
Unknown	1 (7)	1 (3)	2 (10)	0	0	0	0
BMI, median (range), kg/m^2^	25.6 (19.8–33.8)	25.8 (18.5–33.6)	25.2 (17–35.7)	26.3 (21.6–36.3)	25.8 (18.2–35.9)	28.7 (23.5–28.8)	26.5 (15.6–33.5)
ECOG performance status, *n* (%)	​	​	​	​	​	​	​
0	7 (47)	13 (37)	3 (15)	5 (45)	1 (11)	1 (33)	1 (11)
1	8 (53)	21 (60)	15 (75)	6 (55)	8 (89)	2 (67)	7 (78)
2	0	1 (3)	2 (10)	0	0	0	0
Missing	0	0	0	0	0	0	1 (11)

Abbreviations: BMI, body mass index; Q1/3W, dosing on day 1 of repeated 21-day cycles; Q2/3W, dosing on days 1 and 8 of repeated 21-day cycles.

### Efficacy

Efficacy results by dosing schedule and cancer type are shown in Table 2 (BICR) and Supplementary Table S4 (investigator assessment). Swimmer plots are shown in Supplementary Fig. S3 (BICR) and Supplementary Fig. S4 (investigator assessment). The confirmed ORRs per either BICR or investigator assessment were 1% (95% CI, 0%–8%) with 1 partial response in participants receiving Q1/3W dosing and 0% in participants receiving Q2/3W dosing. No confirmed complete responses were observed. The confirmed partial response occurred in a participant with HR+/HER2− breast cancer with liver and bone metastases at screening, an ECOG performance status of 0, and 4 prior lines of therapy (including a cyclin-dependent kinase 4/6 inhibitor, hormonal therapy, and chemotherapy); TTR was 1.9 months per both BICR and investigator assessment, and OS was 24 months. Per investigator assessment, this participant had a DOR of 4.2 months and TTF and PFS of 6.0 months but was censored per BICR assessment (BICR considered this participant to have had an ongoing response at the last scan before data cutoff).

**Table 2.** Summary of efficacy results^a^.

​	Q1/3W			Q2/3W			
	TNBC (*n* = 15)	HR+/HER2−(*n* = 35)	NSCLC (*n* = 20)	TNBC (*n* = 11)	NSCLC (*n* = 9)	Ovarian (*n* = 3)	Pancreatic (*n* = 9)
Confirmed ORR (95% CI), %	0	3 (0–15)	0	0	0	0	0
BOR, *n* (%)	​	​	​	​	​	​	​
CR	0	0	0	0	0	0	0
PR	0	1 (3)	0	0	0	0	0
SD	7 (47)	14 (40)	7 (35)	5 (45)	4 (44)	0	0
PD	3 (20)	13 (37)	7 (35)	6 (55)	2 (22)	1 (33)	6 (67)
Not evaluable	3 (20)	2 (6)	0	0	1 (11)	2 (67)	1 (11)
No assessment	2 (13)	5 (14)	6 (30)	0	2 (22)	0	2 (22)
PFS, median (95% CI), months	3.8 (1.4–NA)	3.1 (1.9–4.2)	2.2 (1.5–4.6)	2.1 (1.4–4.2)	4.3 (1.6–NA)	1.9 (1.3–NA)	1.7 (1.4–NA)
TTF, median (95% CI), months	3.8 (1.2–NA)	3.1 (1.9–4.2)	2.2 (1.4–4.6)	2.1 (1.4–4.2)	4.3 (1.6–NA)	1.9 (1.3–NA)	1.7 (1.4–NA)
OS, median (95% CI), months	9.5 (3.6–11.6)	10.7 (5.2–16.4)	5.4 (2.9–8.3)	5 (4–NA)	8.9 (1.8–NA)	8.1 (1.3–NA)	5 (1.4–NA)

Abbreviations: BOR, best overall response; CR, complete response; NA, not available; PD, progressive disease; PR, partial response; Q1/3W, dosing on day 1 of repeated 21-day cycles; Q2/3W, dosing on days 1 and 8 of repeated 21-day cycles; SD, stable disease.

a Responses, PFS, and TTF are per RECIST version 1.1 by BICR.

The median PFS per BICR was 2.3 (95% CI, 2.0–4.1) months with Q1/3W dosing and 1.9 (95% CI, 1.7–2.1) months with Q2/3W dosing. The corresponding results per investigator assessment were 2.0 (95% CI, 1.9–2.1) months and 1.9 (95% CI, 1.5–2.1) months, respectively. The median TTF per BICR was 2.2 (95% CI, 1.9–4.1) months in participants receiving Q1/3W dosing and 1.9 (95% CI, 1.7–2.1) months in participants receiving Q2/3W dosing. The corresponding results per investigator assessment were 1.9 (95% CI, 1.9–2.1) months and 1.9 (95% CI, 1.5–2.0) months, respectively. The median OS was 8.3 (95% CI, 5.2–10.3) months with Q1/3W dosing and 5.5 (95% CI, 4.4–11.0) months with Q2/3W dosing. The difference in median OS between the dosing regimens may have been related to the more aggressive cancer types among participants receiving Q2/3W dosing compared with Q1/3W dosing.

### Safety

AEs by dosing schedule and cancer type are shown in Tables 3 and 4. All 102 participants experienced AEs. Serious AEs occurred in 29 participants (41%) receiving Q1/3W dosing and 16 (50%) receiving Q2/3W dosing, and treatment-related AEs occurred in 58 participants (83%) and 27 participants (84%), respectively. Overall, serious AEs considered to be related to the study drug occurred in 16 participants (16%). The most common treatment-related AEs (occurring in ≥20% of participants with either dosing schedule) were fatigue (30% with Q1/3W dosing and 28% with Q2/3W dosing), nausea (27% and 31%, respectively), diarrhea (23% and 13%), alopecia (17% and 22%), decreased neutrophil count (16% and 22%), and increased aspartate aminotransferase (23% and 6%). Grade ≥3 treatment-related AEs were reported in 21 participants (30%) receiving Q1/3W dosing and 12 (38%) receiving Q2/3W dosing. The most common grade ≥3 treatment-related AEs (occurring in ≥10% of participants with either dosing schedule) were decreased neutrophil count (14% with Q1/3W dosing and 16% with Q2/3W dosing) and fatigue (4% and 13%, respectively). Two participants died of possible treatment-related AEs (urinary tract infection in the context of neutropenia leading to urosepsis and renal failure and cardiac arrest in the setting of metastatic disease to the myopericardium). Both participants had HR+/HER2− breast cancer and were receiving Q1/3W dosing.

**Table 3.** Summary of AEs.

​	Q1/3W			Q2/3W			
	TNBC (*n* = 15)	HR+/HER2−(*n* = 35)	NSCLC (*n* = 20)	TNBC (*n* = 11)	NSCLC (*n* = 9)	Ovarian (*n* = 3)	Pancreatic (*n* = 9)
Any AE	15 (100)	35 (100)	20 (100)	11 (100)	9 (100)	3 (100)	9 (100)
Any SAE	7 (47)	13 (37)	9 (45)	5 (45)	4 (44)	2 (67)	5 (56)
Any treatment-related AE	13 (87)	29 (83)	16 (80)	9 (82)	8 (89)	2 (67)	8 (89)
Led to treatment modification^a^	5 (33)	8 (23)	7 (35)	6 (55)	4 (44)	0	2 (22)
Led to treatment discontinuation	2 (13)	2 (6)	1 (5)	0	2 (22)	0	0
Led to death	0	2 (6)	0	0	0	0	0

Data are *n* (%) of participants.

Abbreviations: Q1/3W, dosing on day 1 of repeated 21-day cycles; Q2/3W, dosing on days 1 and 8 of repeated 21-day cycles; SAE, serious AE.

a Interruption or reduction in dose of study drug.

**Table 4.** Most common^a^ treatment-related AEs.

​	Q1/3W			Q2/3W			
	TNBC (*n* = 15)	HR+/HER2−(*n* = 35)	NSCLC (*n* = 20)	TNBC (*n* = 11)	NSCLC (*n* = 9)	Ovarian (*n* = 3)	Pancreatic (*n* = 9)
Any treatment-related AE	13 (87)	29 (83)	16 (80)	9 (82)	8 (89)	2 (67)	8 (89)
Grades 3–5	6 (40)	8 (23)	7 (35)	4 (36)	6 (67)	1 (33)	1 (11)
Fatigue	3 (20)	12 (34)	6 (30)	1 (9)	6 (67)	1 (33)	1 (11)
Grades 3–5	0	1 (3)	2 (10)	0	4 (44)	0	0
Nausea	3 (20)	11 (31)	5 (25)	3 (27)	2 (22)	1 (33)	4 (44)
Grades 3–5	0	1 (3)	0	0	0	0	0
Diarrhea	5 (33)	7 (20)	4 (20)	1 (9)	2 (22)	1 (33)	0
Grades 3–5	0	0	0	0	0	0	0
Alopecia	2 (13)	6 (17)	4 (20)	2 (18)	2 (22)	2 (67)	1 (11)
Grades 3–5	0	0	0	0	0	0	0
Decreased neutrophil count	2 (13)	5 (14)	4 (20)	4 (36)	1 (11)	1 (33)	1 (11)
Grades 3–5	2 (13)	5 (14)	3 (15)	2 (18)	1 (11)	1 (33)	1 (11)
Increased aspartate aminotransferase	3 (20)	8 (23)	5 (25)	1 (9)	1 (11)	0	0
Grades 3–5	0	1 (3)	1 (5)	0	0	0	0
Decreased appetite	1 (7)	4 (11)	7 (35)	0	1 (11)	1 (33)	1 (11)
Grades 3–5	0	0	0	0	0	0	0
Decreased hemoglobin	2 (13)	5 (14)	2 (10)	1 (9)	3 (33)	0	1 (11)
Grades 3–5	0	0	0	0	0	0	0
Pyrexia	4 (27)	5 (14)	0	0	1 (11)	0	1 (11)
Grades 3–5	0	0	0	0	0	0	0
Increased alanine aminotransferase	3 (20)	4 (11)	3 (15)	1 (9)	0	0	0
Grades 3–5	0	0	0	0	0	0	0
Vomiting	0	4 (11)	3 (15)	0	1 (11)	1 (33)	1 (11)
Grades 3–5	0	0	0	0	0	0	0
Peripheral sensory neuropathy	0	6 (17)	1 (5)	1 (9)	0	0	1 (11)
Grades 3–5	0	0	0	0	0	0	0
Decreased blood potassium	1 (7)	1 (3)	1 (5)	2 (18)	1 (11)	2 (67)	0
Grades 3–5	1 (7)	0	1 (5)	2 (18)	0	1 (33)	0
Peripheral neuropathy	1 (7)	0	3 (15)	1 (9)	2 (22)	0	1 (11)
Grades 3–5	0	0	0	0	1 (11)	0	0
Decreased lymphocyte count	0	0	0	0	3 (33)	1 (33)	0
Grades 3–5	0	0	0	0	2 (22)	0	0

Data are *n* (%) of participants. AEs are sorted by overall frequency.

Abbreviations: Q1/3W, dosing on day 1 of repeated 21-day cycles; Q2/3W, dosing on days 1 and 8 of repeated 21-day cycles.

a Occurring in ≥10% of participants with either dosing schedule.

Treatment-related AEs led to dose interruption or reduction of zilovertamab vedotin in 20 participants (29%) receiving Q1/3W dosing and 12 (38%) receiving Q2/3W dosing. The most common treatment-related events leading to dose interruption or reduction (occurring in ≥5% of participants with either dosing schedule) were decreased neutrophil count (1% with Q1/3W dosing and 19% with Q2/3W dosing), fatigue (3% and 9%, respectively), and peripheral neuropathy (3% and 9%). Treatment-related AEs led to permanent discontinuation of zilovertamab vedotin in five participants (7%) receiving Q1/3W dosing and 2 (6%) receiving Q2/3W dosing. Peripheral neuropathy was the only treatment-related AE to result in the permanent discontinuation of study drug in >1 participant [0 with Q1/3W dosing and 2 (6%) with Q2/3W dosing].

### Pharmacokinetics

In participants who received Q1/3W dosing, the mean AUC_0–504h_ was 6,720 hours•µg/mL for total antibody, 4,030 hours•µg/mL for total ADC, and 0.828 hours•µg/mL for MMAE (Supplementary Table S5; Supplementary Figs. S5–S7). The mean C_max_ was 51.2 µg/mL, 53.4 µg/mL, and 0.00393 µg/mL, respectively. The T_max_ of MMAE was delayed in comparison with total ADC (165.43 vs. 2.38 hours). The t_1/2_s for total antibody, total ADC, and MMAE were 6.89, 4.19, and 3.57 days, respectively. The geometric mean ratio of the AUC of total ADC to total antibody was 0.63.

In participants treated with Q2/3W dosing, the mean AUC_0–504h_ was 7,770 hours•µg/mL for total antibody (an approximate 16% increase vs. Q1/3W dosing), 4,900 hours•µg/mL for total ADC (an approximate 22% increase vs. Q1/3W dosing), and 0.684 hours•µg/mL for MMAE (an approximate 17% decrease vs. Q1/3W dosing; Supplementary Table S6; Supplementary Figs. S8–S10). The mean C_max_ of MMAE was lower with Q2/3W dosing (0.00121 µg/mL on day 1, 0.00289 µg/mL on day 8) compared with Q1/3W dosing (0.00393 µg/mL). As observed with Q1/3W dosing, the T_max_ of MMAE on day 8 of cycle 1 was also delayed in comparison with total ADC (142.58 vs. 0.58 hours) with Q2/3W dosing.

### Expression–response relationships

IHC analyses were performed on a subset of 34 non-TNBC participants and 27 participants with NSCLC from the 88 participants who had been treated at the time of evaluation. Participants with TNBC (*n* = 21) were excluded because none were in the longer treatment cohort. Participants with other tumor types (*n* = 6) were excluded because of small numbers at the time of evaluation. Non-TNBC participants and participants with NSCLC who were receiving ongoing treatment for <6 cycles at the time of biospecimen identification, or who discontinued treatment before the first interval tumor assessment, were excluded because treatment cohort could not be assigned (*n* = 30). Of the 31 remaining participants, 19 had tumor blocks available that were selected for processing. Of these 19 biospecimens, 17 (89%) had sufficient tumor content to perform IHC analyses. Eight participants with assessable biospecimens had a short duration of treatment, and nine participants had a long duration of treatment. The mean *H*-score for ROR1 was 6 (95% CI, 0–14), and the median was 0 (range, 0–65). Three participants had non-zero *H*-scores (10, 30, and 65) with no clear difference between the short and longer treated cohorts (Supplementary Table S7).

The mean Ki-67 *H*-score was 84 (95% CI, 57–100), and the median was 80 (range, 0–195). No clear difference in Ki-67 *H*-score between the short and longer treated cohorts was observed [Supplementary Table S7; mean, 79 (short) and 88 (longer)].

## Discussion

Zilovertamab vedotin demonstrated limited antitumor activity in participants with previously treated metastatic solid tumors. During the study, 1 of 70 participants treated with Q1/3W dosing achieved a partial response for a confirmed ORR of 1% (95% CI, 0%–8%) per RECIST version 1.1 as assessed by BICR (primary endpoint). No complete responses were observed. The confirmed ORR among the 32 participants treated with Q2/3W dosing was 0%. Investigator-assessed findings corroborated the BICR results. Zilovertamab vedotin had a manageable safety and tolerability profile, regardless of the dosing schedule, with 16% of participants experiencing treatment-related serious AEs. Pharmacokinetic results suggested potential benefits with Q2/3W dosing compared with Q1/3W dosing.

The low confirmed ORR is in contrast with the results in participants with relapsed or refractory non–Hodgkin lymphoma. In the phase 1, dose-escalation waveLINE-001 study (7, 14), the ORR was 37% (95% CI, 22%–53%) with 5 complete responses and 10 partial responses among 41 participants treated with zilovertamab vedotin up to 2.5 mg/kg Q1/3W (7). The median DOR among these 15 responders was 7.8 (range, 2.1–17.6+) months (7). No responses were seen in participants with chronic lymphocytic leukemia or small lymphocytic lymphoma (*n* = 7) or acute myeloid leukemia (*n* = 3; ref. 14). Preliminary results from the phase 2 waveLINE-004 study indicated an ORR of 30% (95% CI, 12%–54%), with 2 complete responses and 4 partial responses among 20 evaluable participants with relapsed or refractory diffuse large B-cell lymphoma (15). All participants in that study were treated with zilovertamab vedotin 2.5 mg/kg Q1/3W. Correlations with ROR1 protein expression were not reported in either study. Differences in antitumor activity between our study and the previous studies may be related to the cancer types enrolled. Our study included cancer types that are among the most aggressive and difficult to treat. Five-year survival rates upon diagnosis of metastatic disease are only approximately 3% for pancreatic cancer, 8% for lung cancer, and 30% for breast and ovarian cancers compared with 70% for non–Hodgkin lymphoma (17). ADC responses to solid and hematologic malignancies vary based on target expression, tissue penetration, and tumor lineage.

The types of AEs observed in our study were consistent with those reported in participants with non–Hodgkin lymphoma (14, 15) and with other vedotin-based ADCs (18, 19). In our study, the most common treatment-related AEs were fatigue (29% overall), nausea (28%), and diarrhea (20%). Small differences between the dosing regimens were observed in the proportions of participants who experienced serious AEs (50% with Q2/3W dosing vs. 41% with Q1/3W dosing), grade ≥3 treatment-related AEs (38% vs. 30%), and treatment-related AEs leading to interruption or dose reduction (38% vs. 29%). Rates of AEs leading to interruption or dose reduction were comparable to those observed with other ADCs in participants with solid tumors, including trastuzumab deruxtecan (20), sacituzumab govitecan (21), and the investigational agent YL201 (22). In the study of trastuzumab deruxtecan, treatment-related AEs led to interruption or dose reduction in 85 of 267 (32%) participants (20). Rates of permanent discontinuation of zilovertamab vedotin in our study were similar in participants receiving Q1/3W dosing (7%) compared with Q2/3W dosing (6%), suggesting manageable toxicity with both dosing schedules. Two treatment-related AEs were fatal, as described previously.

Pharmacokinetic analyses showed that Q2/3W dosing, compared with Q1/3W dosing, resulted in higher ADC exposure over the 3-week interval and lower C_max_ and AUC values for MMAE. This suggests that Q2/3W dosing has the potential to maximize target engagement via ADC binding to ROR1-expressing tumor cells without increasing MMAE-related toxicities. Additionally, Q2/3W dosing is advantageous considering the short t_1/2_ of ADC seen with Q1/3W dosing. The C_max_ of MMAE associated with Q2/3W dosing in our study was lower than (23, 24) or similar to (25) those reported with the approved doses of other vedotin-based ADCs.

Translational studies were limited by the small sample size with available tissue. In participants with samples available, we did not identify expression–response relationships associated with either ROR1 or Ki-67 protein expression as measured by IHC and clinical outcomes. It is notable that the trial did not select for ROR1 expression and that ROR1 expression was noted in only 3 of 17 participants with adequate tissue. This is consistent with recent data indicating that many tumor types have low prevalence of ROR1 expression, whereas in some others, such as mesothelioma, it is more frequent (26). Prospectively selecting participants or tumor subtypes for ROR1 expression could result in higher antitumor activity.

A limitation of our study was the small sample size, particularly for the ovarian and pancreatic cohorts. The pharmacokinetic differences seen with Q1/3W versus Q2/3W dosing did not seem to translate into any apparent differences in efficacy or safety in our study, although tumor types varied substantially between dosing schedules. All participants were treated with monotherapy and had previously treated metastatic disease. It is unknown whether initiating zilovertamab vedotin earlier or combining it with complementary agents might have improved antitumor activity.

Our study of zilovertamab vedotin monotherapy reports on an ADC targeting ROR1 for the treatment of participants with advanced solid tumors. In addition, ongoing phase 2/3 studies of zilovertamab vedotin are assessing monotherapy and combination therapy approaches in participants with diffuse large B-cell lymphoma [NCT05139017 (27) and NCT05406401 (28) and select aggressive and indolent B-cell malignancies NCT05458297 (29)]. Other first-in-human studies evaluating ADCs targeting ROR1 for the treatment of participants with advanced solid tumors have been initiated (30, 31). The NBE-002 study [NCT04441099 (30)] was stopped early after enrolling 12 of a planned 100 participants. The sponsor terminated the program, and the results have yet to be presented or published. In this regard, it is important to note that NBE-002 includes the potent anthracycline derivative PNU-159682 as its cytotoxic payload in contrast to MMAE for zilovertamab vedotin (32). Further study is needed to determine whether the cytotoxic payload rather than targeting ROR1 is responsible for any associated ADC toxicity. Choice of cytotoxic payload is likely to play an important role in the safety profile of ADCs (33). The CS5001 study [NCT05279300 (30)] is currently recruiting and has a planned enrollment of 156 participants with advanced solid tumors and lymphomas. The results are expected in 2025.

In conclusion, zilovertamab vedotin had minimal antitumor activity, with only a single responder in participants with previously treated metastatic solid tumors of various histologic subtypes. Safety was generally manageable with dose interruption or reduction. The results suggest that further development of zilovertamab vedotin in these solid tumors is not warranted.

## Supplementary Material

Supplemental Fig S1 Supplemental Fig S1. Study Design

Supplemental Fig S2 Supplemental Fig S2. Participant Disposition

Supplemental Fig S3 Supplemental Fig S3. Swimlane Plots for Response Events and Disposition During Study Treatment per BICR

Supplemental Fig S4 Supplemental Fig S4. Swimlane Plots for Response Events and Disposition During Study Treatment by Investigator

Supplemental Fig S5 Supplemental Fig S5. Plasma Conc. vs Time Profiles of Total AB in C1 Following Zilovertamab Vedotin IV Once Every 3 Wks

Supplemental Fig S6 Supplemental Fig S6. Plasma Conc. vs Time Profiles of Total ADC in C1 Following Zilovertamab Vedotin IV Once Every 3 Wks

Supplemental Fig S7 Supplemental Fig S7. Plasma Conc. vs Time Profiles of MMAE in C1 Following Zilovertamab Vedotin IV Once Every 3 Wks

Supplemental Fig S8 Supplemental Fig S8. Plasma Conc. vs Time Profiles of Total AB in C1 Following Zilovertamab Vedotin IV Twice Every 3 Wks

Supplemental Fig S9 Supplemental Fig S9. Plasma Conc. vs Time Profiles of Total ADC in C1 Following Zilovertamab Vedotin IV Twice Every 3 Wks

Supplemental Fig S10 Supplemental Fig S10. Plasma Conc. vs Time Profiles of MMAE in C1 Following Zilovertamab Vedotin IV Twice Every 3 Wks

Supplemental Table S1 Supplemental Table S1. Dose Reduction Levels for the Twice Every 3 Weeks Dosing Schedule

Supplemental Table S2 Supplemental Table S2. Representativeness of the Study Population

Supplemental Table S3 Supplemental Table S3. Study Drug Exposure and Follow-Up Duration

Supplemental Table S4 Supplemental Table S4. Efficacy Results per RECIST Version 1.1 by Investigator Assessment

Supplemental Table S5 Supplemental Table S5. Geometric Mean Plasma PK Parameter Values in C1 Following Zilovertamab Vedotin IV Once Every 3 Wks

Supplemental Table S6 Supplemental Table S6. Geometric Mean Plasma PK Parameter Values in C1 Following Zilovertamab Vedotin IV Twice Every 3 Wks

Supplemental Table S7 Supplemental Table S7. ROR1 and Ki-67 IHC Expression for Participants With Short and Longer Durations of Treatment
