# Supplementary material for: Phase 2 Study of Zilovertamab Vedotin in Participants with Metastatic Solid Tumors
Source: Cancer Res Commun. 2025 Sep 17;5(9):1664–73. doi: 10.1158/2767-9764.CRC-25-0019 (PMC12442023; doi:10.1158/2767-9764.CRC-25-0019)

**Supplemental Figure S1.** Study design. HR+/HER2−, hormone receptor‒positive/human epidermal growth factor receptor 2‒negative breast cancer; NSCLC, non‒small-cell lung cancer; Q1/3W, dosing on day 1 of repeated 21-day cycles; Q2/3W, dosing on days 1 and 8 of repeated 21-day cycles; TNBC, triple-negative breast cancer.


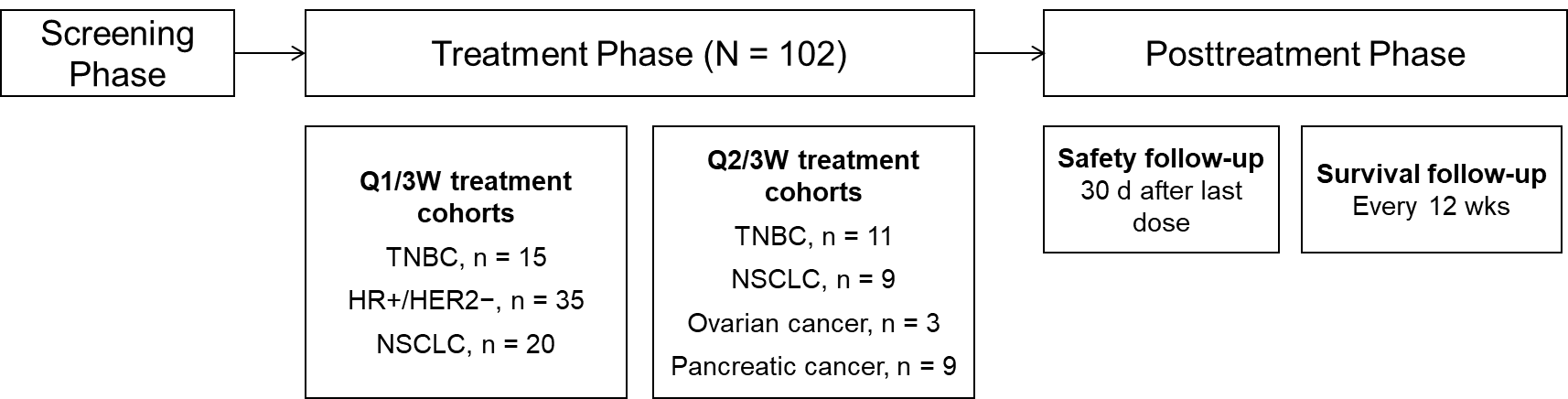

Supplement: Supplemental Fig S1 — Study Design [file crc-25-0019_supplemental_fig_s1_suppsf1.docx]
