# Supplementary material for: Phase 2 Study of Zilovertamab Vedotin in Participants with Metastatic Solid Tumors
Source: Cancer Res Commun. 2025 Sep 17;5(9):1664–73. doi: 10.1158/2767-9764.CRC-25-0019 (PMC12442023; doi:10.1158/2767-9764.CRC-25-0019)

**Supplemental Figure S4.** Swimlane plots for response events and disposition during study treatment by investigator assessment for (A) Q1/3W and (B) Q2/3W. CR, complete response; HR+/HER2−, hormone receptor‒positive/human epidermal growth factor receptor 2‒negative breast cancer; NE, not evaluable; NSCLC, non‒small-cell lung cancer; PD, progressive disease; PR, partial response; Q1/3W, dosing on day 1 of repeated 21-day cycles Q2/3W, dosing on days 1 and 8 of repeated 21-day cycles; SD, stable disease; TNBC, triple-negative breast cancer.

A.

**
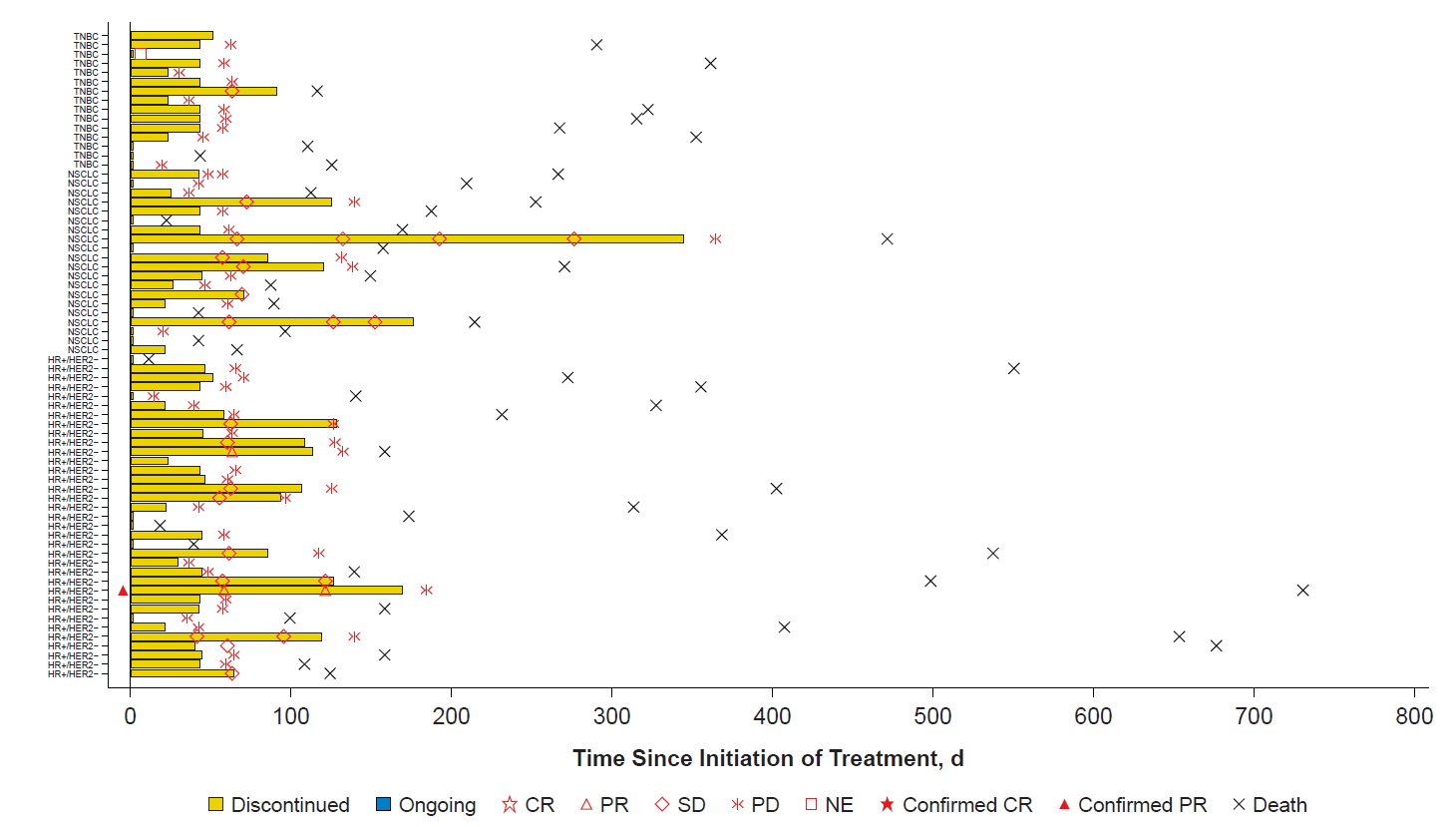
**

B.


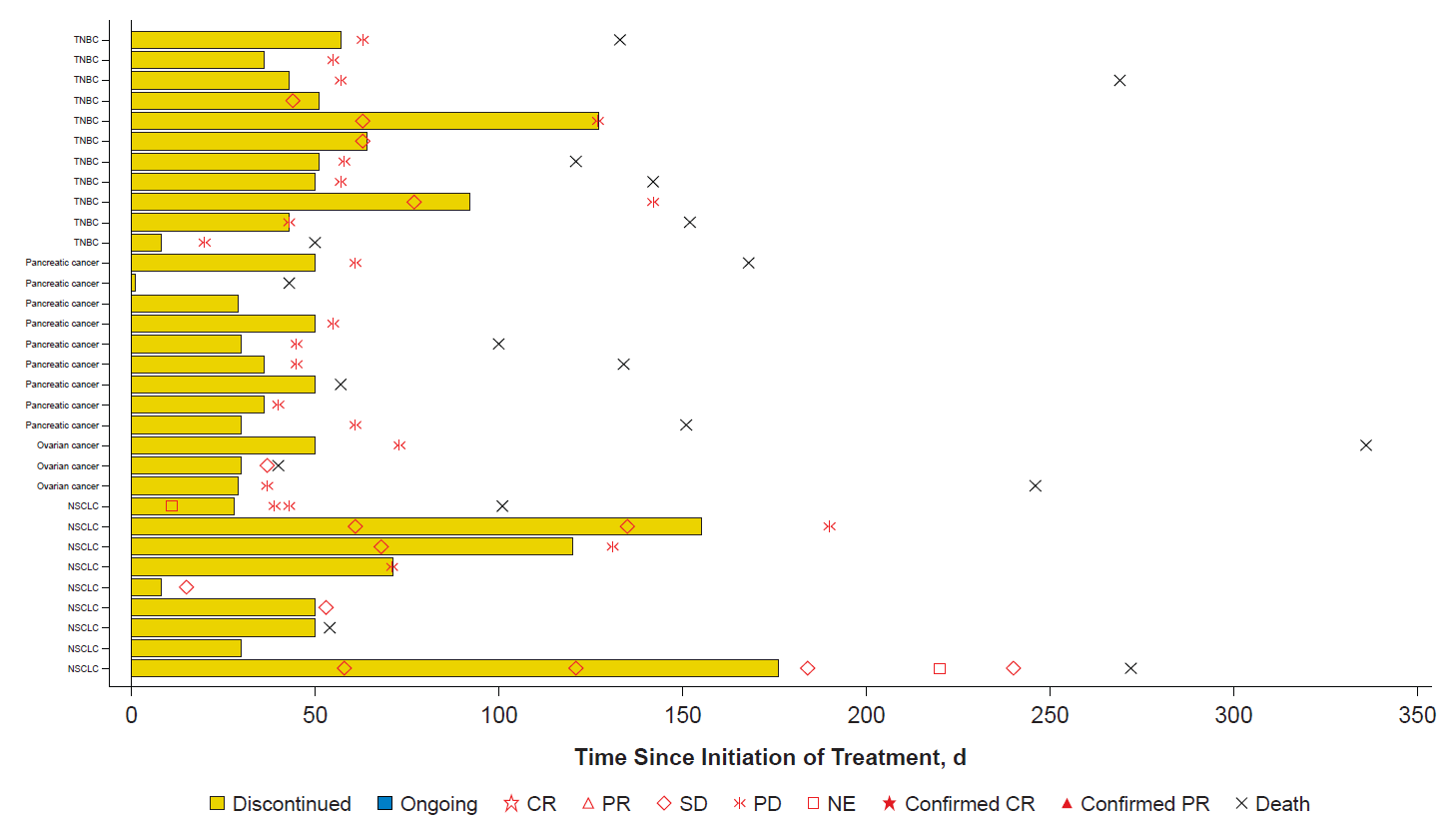

Supplement: Supplemental Fig S4 — Swimlane Plots for Response Events and Disposition During Study Treatment by Investigator [file crc-25-0019_supplemental_fig_s4_suppsf4.docx]
