# Supplementary material for: Phase 2 Study of Zilovertamab Vedotin in Participants with Metastatic Solid Tumors
Source: Cancer Res Commun. 2025 Sep 17;5(9):1664–73. doi: 10.1158/2767-9764.CRC-25-0019 (PMC12442023; doi:10.1158/2767-9764.CRC-25-0019)

**Supplemental Figure S7.** Plasma concentration versus time profiles of MMAE in cycle 1 following intravenous infusion of zilovertamab vedotin 2.5 mg/kg Q1/3W. Data are plotted on (A) linear and (B) semi-log scales. N values range from 51 to 70 across the time points. MMAE, monomethyl auristatin E; Q1/3W, dosing on day 1 of repeated 21-day cycles; SD, standard deviation.

A.


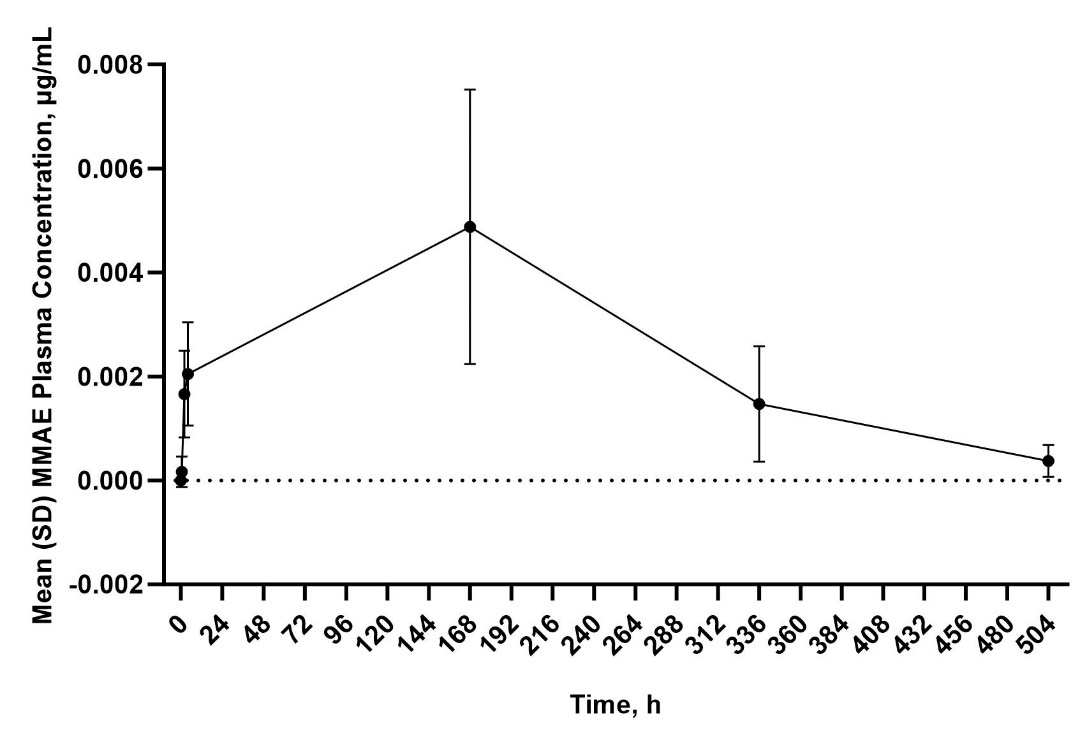


B.


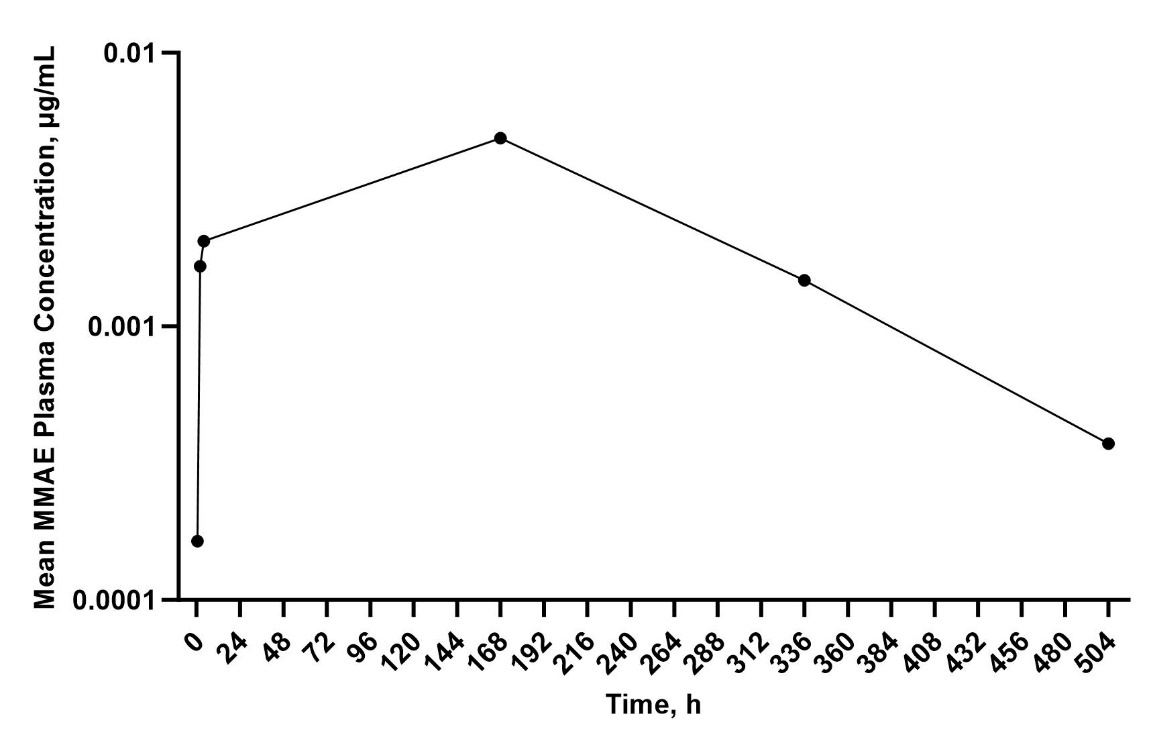

Supplement: Supplemental Fig S7 — Plasma Conc. vs Time Profiles of MMAE in C1 Following Zilovertamab Vedotin IV Once Every 3 Wks [file crc-25-0019_supplemental_fig_s7_suppsf7.docx]
