# Supplementary material for: Phase 2 Study of Zilovertamab Vedotin in Participants with Metastatic Solid Tumors
Source: Cancer Res Commun. 2025 Sep 17;5(9):1664–73. doi: 10.1158/2767-9764.CRC-25-0019 (PMC12442023; doi:10.1158/2767-9764.CRC-25-0019)
Supplement: Supplemental Table S1 — Dose Reduction Levels for the Twice Every 3 Weeks Dosing Schedule [file crc-25-0019_supplemental_table_s1_suppst1.docx]

## Supplemental Table S1. Dose Reduction Levels for the Q2/3W Dosing Schedule

| **Dose Level, mg/kg** | Dose Level Description |
| --- | --- |
| 1.0 | Dose reduction levels |
| 1.25 |  |
| 1.5 | Dose reduction level or starting dose in participants who require coadministration of a strong inhibitor of CYP3A4 |
| 1.75 | Starting dose (in the absence of a strong inhibitor of CYP3A4) |

CYP3A4, cytochrome P450 3A4; Q2/3W, dosing on days 1 and 8 of repeated 21-day cycles.
