# Supplementary material for: Phase 2 Study of Zilovertamab Vedotin in Participants with Metastatic Solid Tumors
Source: Cancer Res Commun. 2025 Sep 17;5(9):1664–73. doi: 10.1158/2767-9764.CRC-25-0019 (PMC12442023; doi:10.1158/2767-9764.CRC-25-0019)
Supplement: Supplemental Table S2 — Representativeness of the Study Population [file crc-25-0019_supplemental_table_s2_suppst2.docx]

## Supplemental Table S2. Representativeness of the Study Population

| **Cancer Type** | **Advanced or Metastatic Solid Tumors** |
| --- | --- |
| Considerations related to |  |
| Sex | The incidence of cancer at any site in males and females the US (2017–2021) was 478.7/100,000 and 416.7/100,000, respectively. |
| Age | In the US (2017–2021), the median age at diagnosis of cancer at any site was 67 years, with most diagnoses occurring in the 65–74 years (30.2%) age bracket. |
| Race/ethnicity | In males in the US (2017–2021), incidence of cancer at any site was highest in non-Hispanic Black males (526.5/100,000) followed by non-Hispanic White males (510.7/100,000), and non-Hispanic American Indian/Alaska Native males (442.2/100,000). In females, the incidence of cancer at any site was highest in non-Hispanic White females (447.0/100,000), non-Hispanic American Indian/Alaska Native females (421.1/100,000), and non-Hispanic Black females (403.1/100,000). |
| Geography | The incidence of new cancer cases in the US (2022) was 367.0/100,000, with a mortality rate of 82.3/100,000; in Canada (2022), incidence of new cancer cases was 345.9/100,000, with a mortality rate of 96.4/100,000. |
| Overall representativeness of this study | Participants in our study were enrolled at multiple sites across North America. The study had a higher proportion of female (86%) than male participants; this is likely due to some of the tumor types occurring exclusively or predominantly in females (ovarian cancer, TNBC, and HR+/HER2− breast cancer). Median age was 60 (range, 32‒88) years, with 38% of participants aged ≥65 years. Study participants were predominantly White (74%), which aligns with the US demographic (75% White). Overall, the demographic and baseline characteristics in our study population are broadly consistent with that reported in the literature. |

HR+/HER2−, hormone receptor‒positive/human epidermal growth factor receptor 2‒negative; TNBC, triple-negative breast cancer.
