# Supplementary material for: Phase 2 Study of Zilovertamab Vedotin in Participants with Metastatic Solid Tumors
Source: Cancer Res Commun. 2025 Sep 17;5(9):1664–73. doi: 10.1158/2767-9764.CRC-25-0019 (PMC12442023; doi:10.1158/2767-9764.CRC-25-0019)
Supplement: Supplemental Table S3 — Study Drug Exposure and Follow-Up Duration [file crc-25-0019_supplemental_table_s3_suppst3.docx]

Supplemental Table S3. Study Drug Exposure and Follow-Up Duration

|  | **Q1/3W** | | | **Q2/3W** | | | |
| --- | --- | --- | --- | --- | --- | --- | --- |
|  | TNBC  (n = 15) | **HR+/HER2−**  **(n = 35)** | **NSCLC**  **(n = 20)** | **TNBC**  **(n = 11)** | **NSCLC**  **(n = 9)** | **Ovarian**  **(n = 3)** | **Pancreatic**  **(n = 9)** |
| Duration of therapy, median (range), mo | 1.4 (0.0–3.0) | 1.4 (0.0–5.6) | 1.1 (0.0–11.3) | 1.7 (0.3–4.2) | 1.6 (0.3–5.8) | 1.0 (1.0–1.6) | 1.2 (0.0–1.6) |
| Doses, median (range), n | 2 (1–5) | 3 (1–9) | 2 (1–11) | 4 (2–12) | 4 (1–18) | 4 (4–6) | 4 (1–6) |
| Cycles, median (range), n | 3 (1–5) | 3 (1–9) | 2.5 (1–16) | 3 (1–6) | 3 (1–9) | 2 (2–3) | 2 (1–3) |
| Duration of follow-up, median (range), mo | 4.1 (0.9–17.3) | 8.9 (0.4–27.5) | 5.4 (0.7–29.2) | 4.7 (1.6–14.3) | 7.4 (0.8–10.6) | 8.1 (1.3–11.0) | 4.4 (1.4–9.2) |

HR+/HER2−, hormone receptor‒positive/human epidermal growth factor receptor 2‒negative breast cancer; NSCLC, non‒small-cell lung cancer; Q1/3W, dosing on day 1 of repeated 21-day cycles; Q2/3W, dosing on days 1 and 8 of repeated 21-day cycles; TNBC, triple-negative breast cancer.
