# Supplementary material for: Phase 2 Study of Zilovertamab Vedotin in Participants with Metastatic Solid Tumors
Source: Cancer Res Commun. 2025 Sep 17;5(9):1664–73. doi: 10.1158/2767-9764.CRC-25-0019 (PMC12442023; doi:10.1158/2767-9764.CRC-25-0019)
Supplement: Supplemental Table S4 — Efficacy Results per RECIST Version 1.1 by Investigator Assessment [file crc-25-0019_supplemental_table_s4_suppst4.docx]

Supplemental Table S4. Efficacy Results per Response Evaluation Criteria in Solid Tumors Version 1.1 by Investigator Assessment

|  | **Q1/3W** | | | **Q2/3W** | | | |
| --- | --- | --- | --- | --- | --- | --- | --- |
|  | TNBC  (n = 15) | **HR+/HER2−**  **(n = 35)** | **NSCLC**  **(n = 20)** | **TNBC**  **(n = 11)** | **NSCLC**  **(n = 9)** | **Ovarian**  **(n = 3)** | **Pancreatic**  **(n = 9)** |
| Confirmed ORR (95% CI), % | 0 | 3 (0–15) | 0 | 0 | 0 | 0 | 0 |
| BOR, n (%) |  |  |  |  |  |  |  |
| CR | 0 | 0 | 0 | 0 | 0 | 0 | 0 |
| PR | 0 | 1 (3) | 0 | 0 | 0 | 0 | 0 |
| SD | 1 (7) | 10 (29) | 6 (30) | 4 (36) | 4 (44) | 0 | 0 |
| PD | 10 (67) | 19 (54) | 9 (45) | 7 (64) | 2 (22) | 2 (67) | 6 (67) |
| Not evaluable | 1 (7) | 0 | 0 | 0 | 1 (11) | 1 (33) | 0 |
| No assessment | 3 (20) | 5 (14) | 5 (25) | 0 | 2 (22) | 0 | 3 (33) |
| PFS, median (95% CI), mo | 1.9 (1.2–2.1) | 2.1 (1.9–3.2) | 2.0 (1.4–4.5) | 1.9 (1.4–4.2) | 4.3 (1.3–NA) | 1.3 (1.2–NA) | 1.6 (1.3–NA) |
| TTF, median (95% CI), mo | 1.8 (1.0–1.9) | 2.1 (1.9–3.2) | 2.0 (1.4–4.5) | 1.9 (1.4–4.2) | 2.3 (1.3–6.2) | 1.3 (1.2–NA) | 1.6 (1.3–NA) |

BOR, best overall response; CR, complete response; HR+/HER2−, hormone receptor‒positive/human epidermal growth factor receptor 2‒negative breast cancer; NA, not available; NSCLC, non−small-cell lung cancer; ORR, objective response rate; OS, overall survival; PD, progressive disease; PFS, progression-free survival; PR, partial response; Q1/3W, dosing on day 1 of repeated 21-day cycles; Q2/3W, dosing on days 1 and 8 of repeated 21-day cycles; SD, stable disease; TNBC, triple-negative breast cancer; TTF, time to treatment failure.
