# Supplementary material for: Phase 2 Study of Zilovertamab Vedotin in Participants with Metastatic Solid Tumors
Source: Cancer Res Commun. 2025 Sep 17;5(9):1664–73. doi: 10.1158/2767-9764.CRC-25-0019 (PMC12442023; doi:10.1158/2767-9764.CRC-25-0019)
Supplement: Supplemental Table S5 — Geometric Mean Plasma PK Parameter Values in C1 Following Zilovertamab Vedotin IV Once Every 3 Wks [file crc-25-0019_supplemental_table_s5_suppst5.docx]

## Supplemental Table S5. Geometric Mean Plasma Pharmacokinetic Parameter Values in Cycle 1 Following Intravenous Infusion of Zilovertamab Vedotin 2.5 mg/kg Q1/3W

|  | **AUC_0–504h_,**  **h∙µg/mL** | **AUC_last_,**  **h∙µg/mL** | **C_max_,**  **µg/mL** | **T_max_,**  **h^a^** | **t_1/2_,**  **d** | **GMR of Total ADC/Total Antibody^b^** |
| --- | --- | --- | --- | --- | --- | --- |
| Total antibody (N = 70) | 6720 (28.6)  (n = 62) | 5770 (84.9) | 51.2 (23.3) | 2.42 (0.50–5.75) | 6.89 (32.2)  (n = 62) | 0.63 (15.2) |
| Total ADC (N = 70) | 4030 (27.3)  (n = 62) | 3620 (71.2) | 53.4 (20.5) | 2.38 (0.50–4.52) | 4.19 (31.1)  (n = 61) |  |
| MMAE (N = 70) | 0.828 (58.1)  (n = 28) | 0.766 (130.7) | 0.00393 (65.7) | 165.43 (4.25–215.00) | 3.57 (7.6)  (n = 2) |  |

Data are geometric means (% geometric coefficient of variation) unless otherwise specified.

ADC, antibody‒drug conjugate; AUC, area under the curve; C_max_, maximum plasma concentration; GMR, geometric mean ratio; MMAE, monomethyl auristatin E; Q1/3W, dosing on day 1 of repeated 21-day cycles; t_1/2_, half-life; T_max_, time to maximum plasma concentration.

^a^Median (minimum–maximum).

^b^Defined as AUC_last_ for total ADC/AUC_last_ for total antibody.
