# Supplementary material for: Phase 2 Study of Zilovertamab Vedotin in Participants with Metastatic Solid Tumors
Source: Cancer Res Commun. 2025 Sep 17;5(9):1664–73. doi: 10.1158/2767-9764.CRC-25-0019 (PMC12442023; doi:10.1158/2767-9764.CRC-25-0019)
Supplement: Supplemental Table S6 — Geometric Mean Plasma PK Parameter Values in C1 Following Zilovertamab Vedotin IV Twice Every 3 Wks [file crc-25-0019_supplemental_table_s6_suppst6.docx]

## Supplemental Table S6. Geometric Mean Plasma Pharmacokinetic Parameter Values in Cycle 1 Following Intravenous Infusion of Zilovertamab Vedotin 1.75 mg/kg Q2/3W

|  | **Cycle 1, Day 1** | | | | | **Cycle 1, Day 8** | | | | | | **Cycle 1** |
| --- | --- | --- | --- | --- | --- | --- | --- | --- | --- | --- | --- | --- |
|  | **N** | **AUC_0–168h_,**  **h∙µg/mL** | **AUC_last_,**  **h∙µg/mL** | **C_max_,**  **µg/mL** | **T_max_,**  **h^a^** | **N** | **AUC_168–336h_,**  **h∙µg/mL** | **AUC_last_,**  **h∙µg/mL** | **C_max_,**  **µg/mL** | **T_max_,**  **h^a^** | **t_1/2_,**  **d** | **AUC_0–504h_,**  **h∙µg/mL** |
| Total antibody | 23 | 2730 (25.0)  (n = 10) | 2530 (33.5)  (n = 20) | 26.2 (39.8) | 0.58  (0.50–0.97) | 27 | 5890 (48.1)  (n = 11) | 5810 (55.6)  (n = 24) | 39.1 (73.7) | 0.58  (0.35–165.45) | NC^b^ | 7770 (34.4)  (n = 15) |
| Total ADC | 23 | 2070 (26.1)  (n = 10) | 1940 (47.9)  (n = 20) | 28.6 (40.8) | 0.58  (0.50–167.92) | 27 | 2900 (66.0)  (n = 11) | 3020 (59.3)  (n = 24) | 35.0 (90.3) | 0.58  (0.00–165.45) | 31.8^c^  (n = 1) | 4900 (40.8)  (n = 17) |
| MMAE | 23 | 0.202 (101.2)  (n = 10) | 0.177 (90.2)  (n = 20) | 0.00121 (332.3) | 167.42  (0.50–191.02) | 27 | 0.596 (103.3)  (n = 14) | 0.701 (87.8)  (n = 24) | 0.00289 (90.9) | 142.58  (0.00–166.60) | 9.63 (15.1)  (n = 3) | 0.684 (109.9)  (n = 15) |

Data are geometric means (% geometric coefficient of variation) unless otherwise specified.

ADC, antibody‒drug conjugate; AUC, area under the curve; C_max_, maximum plasma concentration; MMAE, monomethyl auristatin E; NC, not calculated; Q2/3W, dosing on days 1 and 8 of repeated 21-day cycles; t_1/2_, half-life; T_max_, time to maximum plasma concentration.

^a^Median (minimum–maximum).

^b^Half-life was not calculated due to insufficient data during the terminal phase.

^c^% geometric coefficient of variation was not calculated as data were available for 1 participant.
