# Supplementary material for: Phase 2 Study of Zilovertamab Vedotin in Participants with Metastatic Solid Tumors
Source: Cancer Res Commun. 2025 Sep 17;5(9):1664–73. doi: 10.1158/2767-9764.CRC-25-0019 (PMC12442023; doi:10.1158/2767-9764.CRC-25-0019)
Supplement: Supplemental Table S7 — ROR1 and Ki-67 IHC Expression for Participants With Short and Longer Durations of Treatment [file crc-25-0019_supplemental_table_s7_suppst7.docx]

## Supplemental Table S7. ROR1 and Ki-67 IHC Expression for Participants With Short and Longer Durations of Treatment

| **Participant Number** | **Study Cohort** | **Cycles Treated** | **Treatment Duration Cohort^a^** | **ROR1 H-Score** | **Ki-67 H-score** |
| --- | --- | --- | --- | --- | --- |
| 1 | HR+/HER2− | 3 | Short | 0 | 55 |
| 2 | HR+/HER2− | 3 | Short | 0 | 80 |
| 3 | HR+/HER2− | 3 | Short | 0 | 15 |
| 4 | HR+/HER2− | 3 | Short | 0 | 65 |
| 5 | HR+/HER2− | 3 | Short | 0 | 190 |
| 6 | HR+/HER2− | 3 | Short | 0 | 55 |
| 7 | HR+/HER2− | 3 | Short | 0 | 85 |
| 8 | NSCLC | 3 | Short | 65 | 85 |
| 9 | NSCLC | 9 | Longer | 10 | 45 |
| 10 | HR+/HER2− | 6 | Longer | 0 | 150 |
| 11 | HR+/HER2− | 9 | Longer | 0 | 115 |
| 12 | NSCLC | 10 | Longer | 30 | 30 |
| 13 | NSCLC | 16 | Longer | 0 | 50 |
| 14 | HR+/HER2− | 6 | Longer | 0 | 195 |
| 15 | HR+/HER2− | 6 | Longer | 0 | 0 |
| 16 | HR+/HER2− | 7 | Longer | 0 | 90 |
| 17 | NSCLC | 6 | Longer | 0 | 115 |

HR+/HER2−, hormone receptor‒positive/human epidermal growth factor receptor 2‒negative breast cancer; IHC, immunohistochemistry; Ki-67, marker of proliferation Ki-67; NSCLC, non−small-cell lung cancer; ROR1, receptor tyrosine kinase–like orphan receptor 1.

^a^The short treatment cohort was defined as participants who progressed at the first interval tumor assessment (9 week on study radiology assessment after 3 cycles of treatment), and the longer treatment cohort was defined as participants who remained on study treatment at least 6 cycles of therapy.
